# Case Report: Malignant transformation of deep infiltrating endometriosis: a report of two cases and literature review

**DOI:** 10.3389/fonc.2026.1709462

**Published:** 2026-06-18

**Authors:** Yue Gu, Xuanxuan Zhao, Yongheng Shi, Zhijun Hu, Qing Liu, Hong Zhu, Peiquan Li, Kaijiang Liu

**Affiliations:** 1 Department of Gynecologic Oncology, Renji Hospital, School of Medicine, Shanghai Jiao Tong University, Shanghai, China; 2 Department of Pathology, Renji Hospital, School of Medicine, Shanghai Jiao Tong University, Shanghai, China

**Keywords:** deep infiltrating endometriosis, diagnosis, endometrioid adenocarcinoma, oncology, treatment

## Abstract

Deep infiltrating endometriosis (DIE) is a severe form of endometriosis that rarely undergoes malignant transformation, and when it does, diagnosis is often difficult. Here, we report two cases of long-standing DIE that progressed to endometrioid adenocarcinoma. The first case involved a 44-year-old woman with pelvic DIE complicated by hydronephrosis, who underwent radical surgery with ureteroneocystostomy. Postoperative pathology confirmed well-differentiated endometrioid adenocarcinoma, and she remained disease-free after receiving chemotherapy and targeted therapy. The second case was a 47-year-old woman with an 18-year history of dysmenorrhea and chronic pelvic pain, also complicated by hydronephrosis. Surgical exploration revealed extensive pelvic adhesions and DIE lesions, and pathology confirmed endometrioid adenocarcinoma. She received postoperative chemotherapy, including an antibody-drug conjugate, and showed no evidence of recurrence during follow-up. A review of the literature identified 57 additional reported cases of malignant transformation arising from DIE, most commonly involving the rectosigmoid colon, vagina, and ureter. Endometrioid adenocarcinoma was the most frequently observed histological subtype. Due to its nonspecific clinical manifestations and overlap with benign DIE, diagnosis remains challenging. Current evidence supports a multidisciplinary approach combining complete surgical resection with adjuvant therapy. Clinicians should remain aware of the rare but possible malignant potential of DIE to facilitate earlier diagnosis and appropriate management.

## Introduction

Endometriosis (EMS), defined as the presence of endometrial-like tissue outside the uterine cavity, affects approximately 2–10% of women of reproductive age worldwide, with reported prevalence rising to 30–50% among patients with infertility and chronic pelvic pain (1). Deep infiltrating endometriosis (DIE), characterized by lesions penetrating more than 5 mm beneath the peritoneal surface, frequently involves adjacent organs such as the bowel and ureters. EMS is associated with an increased risk of ovarian cancer, with an overall malignant transformation rate of 0.7%–1%; about 80% of these cases arise from ovarian lesions, most commonly presenting as clear cell carcinoma or endometrioid adenocarcinoma (2). Extraovarian malignant transformation accounts for roughly 20% of cases and mainly involves the rectovaginal septum, colon, and vaginal wall, with endometrioid adenocarcinoma being the predominant histological subtype (3).

Histopathological examination remains the definitive standard for diagnosing malignant transformation arising from endometriosis. The diagnostic criteria were first proposed by Sampson in 1925 (4) and later refined by Scott in 1953 (5). In essence, diagnosis requires several key elements: (1) coexistence of carcinoma and benign endometriotic tissue; (2) a clear histological relationship between the carcinoma and adjacent endometriosis; (3) evidence that the carcinoma originates from the endometriotic focus rather than representing metastasis or secondary invasion; and (4) demonstrable transition from benign to malignant epithelium on microscopic evaluation, including direct continuity between benign and malignant components or visible malignant change within endometriotic lesions (6). However, establishing a pathological diagnosis is particularly challenging in cases of DIE.

In malignant transformation of DIE, approximately 20%–30% of patients show no obvious abnormalities in the ovaries or endometrium on imaging or pathological assessment, with lesions often restricted to deeply infiltrative sites such as the rectovaginal septum and uterosacral ligaments (7). In addition, extensive anatomical distortion caused by progressive DIE further increases surgical difficulty and operative risk (8). Given the limited available evidence, no standardized management strategy has been established for these patients. In this study, we present two cases of malignant transformation arising from DIE and review the relevant literature to improve clinical awareness, with particular attention to hydronephrosis as a presenting feature, molecular profiling using next-generation sequencing, and postoperative adjuvant therapy including antibody–drug conjugates.

## Case representation

### Case one

A 44-year-old woman with a history of right ureteral and pelvic deep infiltrating endometriosis (DIE) underwent laparoscopic surgery in 2018, followed by treatment with a gonadotropin-releasing hormone analogue (GnRH-a). The patient had no family history of endometriosis, ovarian cancer, breast cancer, or Lynch syndrome-associated malignancies. Follow-up showed regular menses with mild dysmenorrhea, accompanied by cyclical rectal tenesmus. One year earlier, routine examination revealed right hydronephrosis, and a ureteral double-J stent was placed. However, subsequent ultrasound indicated progressive hydronephrosis, with intermittent right lumbar discomfort. On physical examination, a fixed right adnexal mass of approximately 3 cm was palpated.

Preoperative evaluation raised strong concern for malignant transformation. Serum tumor markers were markedly elevated, including CA19-9 (264.00 U/mL), CA50 (161.60 IU/mL), CA242 (46.20 U/mL), CA125 (64.60 U/mL), SCC (3.80 ng/mL), and HE4 (271.00 pmol/L), with a premenopausal ROMA score of 83.1%. Pelvic MRI (**Figure 1**) demonstrated a solid mass in the right pelvic floor involving the distal right ureter, showing isointensity on T1WI, slightly hyperintense signal on T2WI, and diffusion restriction on DWI. Contrast-enhanced pelvic CT and CTU showed an ill-defined soft tissue mass adjacent to the cervix, extending to the distal right ureter and resulting in proximal hydronephrosis.

**Figure 1:**
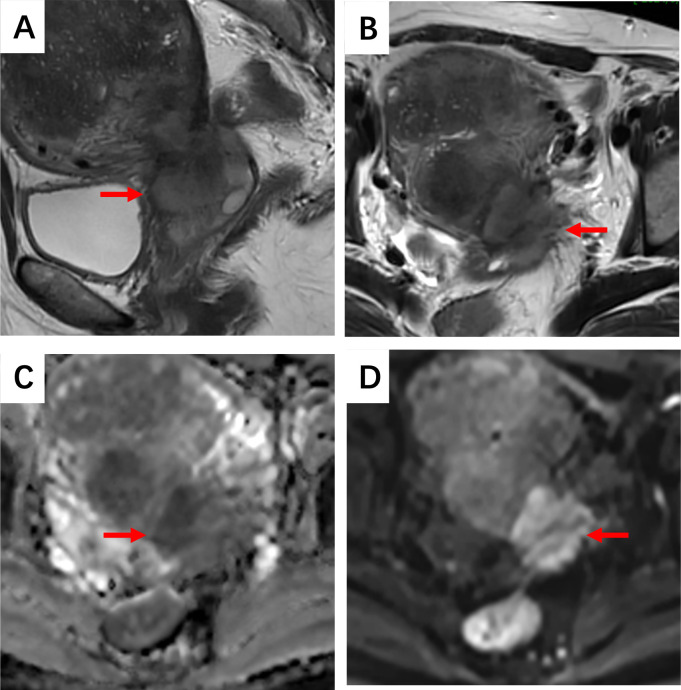
The pelvic MRI demonstrated a soft tissue mass signal in the right pelvic floor (measuring 37×28 mm) shows ill-defined borders with the cervix. The lesion involves the distal right ureter, causing proximal hydroureter [**(A)**, sagittal plane, red arrow). It appears hyperintense on T2-weighted imaging [**(A)**, sagittal plane, red arrow; **(B)**, transverse plane, red arrow), hypointense on diffusion-weighted imaging- apparent diffusion coefficient (DWI-ADC) mapping [**(C)**, transverse plane, red arrow), markedly hyperintense on DWI at b=1000 s/mm² [**(D)**, transverse plane, red arrow].

She underwent laparoscopic radical hysterectomy (TH), bilateral salpingectomy (BS), resection of the anterior rectal wall lesion, right pelvic lymphadenectomy, and right ureteroneocystostomy with placement of a ureteral double-J stent in August 2024. Intraoperatively, a 4 × 5 cm friable tumor was identified in the right pelvis, involving the parametrium, uterosacral ligament, and pelvic sidewall, with encasement of the right ureter. A 1.5 cm reddish lymph node was noted near the right external iliac artery. No ascites or visible peritoneal tumor implants were seen, and both ovaries and fallopian tubes appeared grossly unremarkable. Postoperative pathology confirmed well-differentiated endometrioid adenocarcinoma (FIGO grade 1) involving the anterior rectal wall, right parametrium, pelvic floor, and partial right ureter. The patient met the histopathological criteria for deep infiltrating endometriosis (DIE), with endometriotic tissue extending more than 5 mm beneath the peritoneal surface, and no metastatic involvement of pelvic lymph nodes. Immunohistochemistry (**Figure 2**) showed strong nuclear positivity for PAX-8 (~90%), ER (~90%) and PR (~80%), with patchy P16 expression and a wild-type P53 pattern. Ki-67 was approximately 10–15%. Mismatch repair proteins (MSH2, MSH6, MLH1, PMS2) were intact, WT-1 was negative, and VIM showed cytoplasmic positivity. PD-L1 (22C3) was negative (TPS 0%, CPS 0), and HER2 scored 0. Next-generation sequencing identified somatic mutations in KRAS, PIK3CA, and PALB2, with a tumor mutational burden (TMB) of 1.26 mut/Mb. The patient received six cycles of paclitaxel plus carboplatin combined with bevacizumab, followed by six months of bevacizumab maintenance therapy, and remained disease-free at 14-month follow-up.

**Figure 2:**
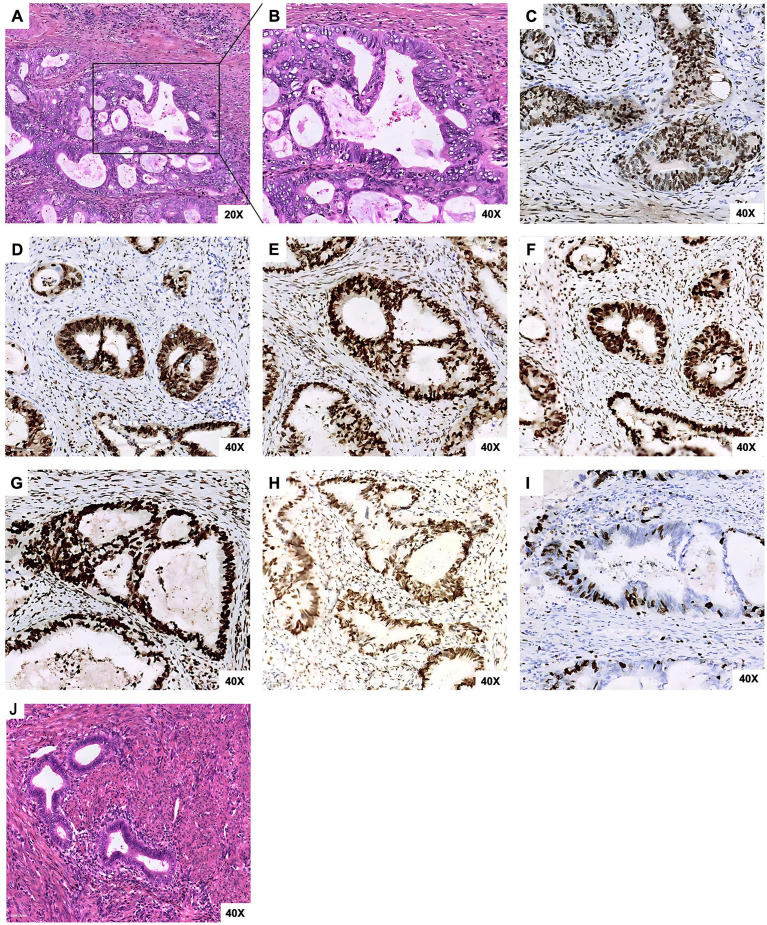
H&E stains and immunohistochemical images for patient 1. Endometrial adenocarcinoma, FIGO grade 1, displaying tubular glandular architecture with focal cribriform fusion and stratified cellular arrangement [H&E, 20×, **(A)**]. Endometrial adenocarcinoma with marked cellular atypia, disorganized growth pattern, loss of nuclear polarity, and identifiable mitotic activity [H&E, 40×, **(B)**]. ER immunohistochemistry demonstrates strong nuclear positivity in tumor cells [IHC, 40×, **(C)**]. PR demonstrates strong nuclear positivity in tumor cells [IHC,40×, **(D)**]. MMR proteins (MLH1, PMS2, MSH2, MSH6) exhibit intact nuclear expression with no evidence of microsatellite instability (MSI) [IHC,40×, **(E-H)**]. Ki-67 demonstrates nuclear positivity in approximately 20% of tumor cells within hotspot regions [IHC,40×, **(I)**]. **(J)** Benign endometriotic glands identified within the same parametrial specimen, confirming the presence of pre-existing deep infiltrating endometriosis (H&E, 40×).

### Case two

A 47-year-old woman presented with an 18-year history of progressive dysmenorrhea and 5 years of lumbosacral pain. Her family history was unremarkable. She had previously received a levonorgestrel-releasing intrauterine system (LNG-IUS), which provided partial symptom relief, but later developed left ureteral calculi with hydronephrosis. Ureteral stenting in September 2024 was followed by aggravation of pelvic pain.

Preoperative workup raised concern for malignant transformation. CA125 (123.00 U/mL) and HE4 (348.00 pmol/L) were elevated, with a premenopausal ROMA score of 90.3%, while other tumor markers were within normal range. On examination, a fixed 3-cm nodule was palpated in the uterosacral ligament region with involvement of the vaginal fornix. Pelvic MRI (**Figure 3**) showed a cervical mass with poorly defined borders extending toward the rectouterine pouch and adjacent cervix, posterior uterine wall, sigmoid colon, and left distal ureter, suggesting possible invasive disease. The lesion appeared isointense on T1WI, slightly hypointense on T2WI with scattered hyperintense foci, and demonstrated heterogeneous enhancement and diffusion restriction.

**Figure 3:**
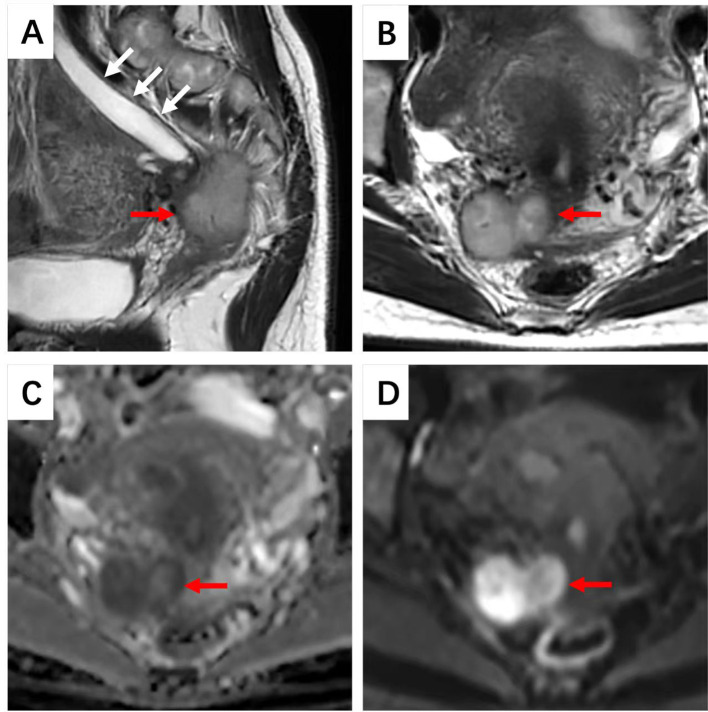
A soft tissue mass in the left lateral cervical wall (approximately 38×42×21 mm) involves the ureter and sigmoid colon was shown in MRI. The lesion exhibits heterogeneous isointense signal on T2-weighted imaging [**(A)**, sagittal plane, red arrow; **(B)**, transverse plane red arrow), marked hypointensity on DWI-ADC mapping [**(C)**, transverse plane red arrow], hyperintensity on DWI at b=1000 s/mm²[**(D)**, transverse plane red arrow].

Intraoperatively, dense pelvic adhesions were encountered. The left parametrium was markedly thickened and firm, and the left adnexa was involved by adhesions between the fallopian tube, ovary, and uterosacral ligament. A right ovarian endometrioma measuring about 3.5 cm was also noted. The rectum was densely adherent to the posterior uterine wall with obliteration of the pouch of Douglas. The patient underwent laparoscopic TH, BS, resection of left parametrium and right uterosacral ligament lesions, resection of rectal anterior and lateral wall lesions, excision of ovarian lesions, and left ureteral double-J stent placement in September 2024.

Pathological examination confirmed well-differentiated endometrioid adenocarcinoma (FIGO grade 1) arising in ectopic endometrial tissue. Deep infiltrating endometriosis was also verified histologically, with endometrial glands and stroma identified more than 5 mm beneath the peritoneal surface. Immunohistochemistry (**Figure 4**) demonstrated hormone receptor positivity, with weak ER expression in approximately 20% of tumor cell nuclei and moderate PR expression in about 80%. The tumor showed a wild-type p53 pattern and patchy weak P16 staining. HER2 was equivocal (2+), and Ki-67 was approximately 20%. CD10 highlighted stromal components but was negative in tumor cells. Mismatch repair proteins were intact. Other markers, including PD-L1 (TPS 0%, CPS <1), GATA-3, and Napsin A, were negative.

**Figure 4:**
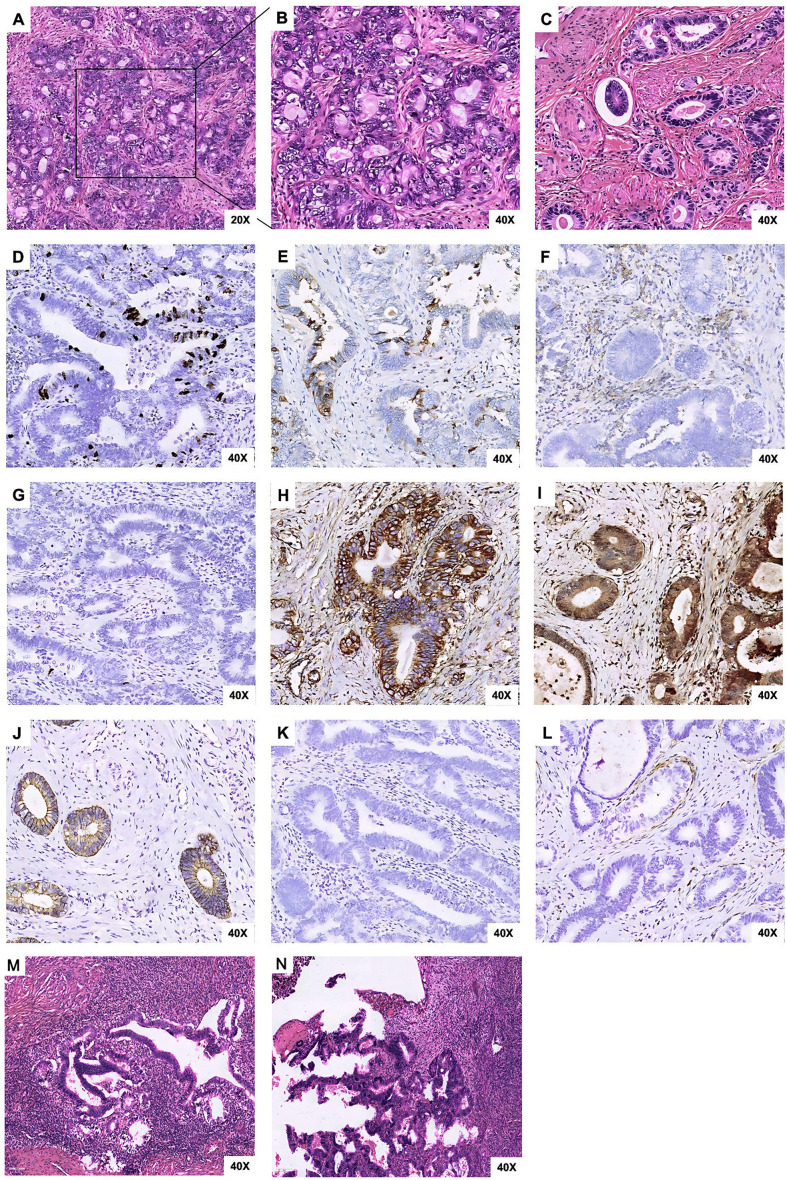
H&E stains and immunohistochemical images for patient 2. Endometrial adenocarcinoma, FIGO grade 1. Invasive glandular proliferation with architectural disarray, composed of tubular glands exhibiting focal cribriform confluence [H&E, 20×, **(A)**]. Endometrial adenocarcinoma demonstrating cribriform glandular fusion, marked cytologic atypia, unequivocal malignant nuclear characteristics, and discernible mitotic activity [H&E, 40×, **(B)**]. Well-differentiated endometrial adenocarcinoma demonstrating infiltrative stromal penetration by well-formed glands with preserved architecture, associated with lymphovascular space invasion [H&E, 40×, **(C)**]. Immunohistochemical (IHC) staining for Ki-67 demonstrates nuclear positivity in approximately 10% of tumor cells within hotspot regions [40×, **(D)**]. P16 demonstrates patchy cytoplasmic positivity [IHC, 40×, **(E)**]. PD-L1 staining is negative in tumor cells with sporadic weak positivity in stromal inflammatory cells (CPS <1, TPS 0%) [IHC, 40×, **(F)**]. P53 expression pattern is wild type, characterized by rare weak nuclear staining of tumor cells and absence of mutant expression [IHC, 40×, **(G)**]. Vimentin demonstrates cytoplasmic positivity in tumor cells [IHC, 40×, **(H)**]. PTEN expression is preserved in tumor cell cytoplasm without evidence of loss [IHC, 40×, **(I)**]. β-Catenin staining localizes to the membranous/cytoplasmic compartment, with no nuclear accumulation observed [IHC, 40×, **(J)**]. Napsin A is negative [IHC, 40×, **(K)**]. CD10 immunostaining is negative in tumor cells [IHC, 40×, **(L)**]. **(M)** Benign deep infiltrating endometriosis identified within the same surgical specimen, showing typical endometrial glands with surrounding stroma (H&E, 40×). **(N)** Serial transition from benign deep infiltrating endometriosis (left) to atypical endometriosis (middle) to endometrioid adenocarcinoma (right), demonstrating the complete morphologic continuum of malignant transformation (H&E, 20×).

Next-generation sequencing identified somatic mutations in KRAS, SPOP, and MUC1, with a tumor mutational burden of 1.26 mut/Mb. The patient subsequently received a carboplatin-based chemotherapy regimen combined with trastuzumab deruxtecan (T-DXd), an anti-HER2 antibody–drug conjugate. At 13-month follow-up, both imaging and tumor marker assessments showed no evidence of disease recurrence. The information about two cases were listed in **Table 1**.

**Table 1:** Summary and comparison of clinical, pathological, and IHC features of the two cases.

Feature	Case 1	Case 2
Clinical features		
Age (years)	44	47
History of DIE (years)	6	18
Chief complaint	Dysmenorrhea, rectal tenesmus, hydronephrosis	Progressive dysmenorrhea, lumbosacral pain, hydronephrosis
CA-125 (U/mL)	64.6	123
HE4 (pmol/L)	271	348
Tumor location	Right parametrium, pelvic floor, right ureter, rectal anterior wall	Left parametrium, left ureter, rectosigmoid, right ovary (endometrioma)
Surgery	TH + BS + rectal lesion resection + right pelvic LND + right ureteroneocystostomy	TH + BS + left parametrial resection + right uterosacral resection + rectal resection + ovarian lesion resection
Pathology		
Histology	Endometrioid adenocarcinoma, FIGO grade 1	Endometrioid adenocarcinoma, FIGO grade 1
Lymph node metastasis	Negative	Not performed
IHC		
ER	90% strong nuclear positive	20% weak nuclear positive
PR	80% strong nuclear positive	80% moderate nuclear positive
P53	Wild-type	Wild-type
Ki-67	10-15%	0.2
MMR status	pMMR (intact)	pMMR (intact)
HER2	0	2+
PD-L1	TPS 0%, CPS 0	TPS 0%, CPS <1
Genetic mutations	KRAS, PIK3CA, PALB2	KRAS, SPOP, MUC1
Adjuvant therapy	Paclitaxel + carboplatin + bevacizumab, then bevacizumab maintenance	Chemotherapy + ADC (anti-HER2)
Follow-up status	No recurrence	No recurrence

## Literature review

To identify reported cases of malignant transformation arising from DIE over the past two decades, we conducted a systematic search of the PubMed database from January 2000 to December 2024. The search strategy combined MeSH terms and keywords including (“deep infiltrating endometriosis” OR “DIE” OR “extraovarian endometriosis” OR “rectovaginal endometriosis” OR “ureteral endometriosis”) with (“malignant transformation” OR “carcinoma” OR “cancer” OR “adenocarcinoma” OR “malignancy”), and was restricted to English-language publications. Eligible studies were those with histologically confirmed malignancy arising from DIE lesions (defined as endometriotic tissue infiltrating more than 5 mm beneath the peritoneal surface), and with available individual patient data in full-text case reports. Reports involving ovarian endometriomas, duplicates, or insufficient clinical/pathological detail were excluded. Two reviewers independently screened all records, with discrepancies resolved by discussion, and reference lists were also checked for completeness. A total of 183 records were identified, and 57 cases from 48 articles were finally included (**Supplementary Table 1**) (9–56).

Across the 57 cases, malignant transformation of DIE was observed in a broad age spectrum, with a mean age of 51 years (range: 25–75). The clinical presentation was heterogeneous but largely non-specific, most commonly including pelvic or abdominal pain, lumbosacral discomfort, dysmenorrhea, vaginal bleeding, hematochezia or rectal bleeding, and pelvic mass. A prior history of endometriosis-related surgery was present in 26 cases (44%), most often total hysterectomy with bilateral salpingo-oophorectomy, followed by ovarian endometrioma excision, BSO alone, unilateral salpingo-oophorectomy, and isolated lesion resections including transvaginal or ureteral procedures. Postoperative management varied considerably, including GnRH-a therapy, hormone replacement therapy after oophorectomy (8/57; mean duration 9 years, range 0.5–19), progestins, danazol, and steroid implantation. The mean interval from initial surgery or diagnosis to malignant transformation was 13.6 years (range: 1–30). Preoperative tumor markers were frequently elevated, particularly CA-125 and HE4, with mean values of 191 U/mL (range: 17–1,048) and 197 pmol/L (range: 104–483), respectively.

Most lesions were confined to the pelvis at diagnosis. Peritoneal dissemination was exceptional, reported in only one case. Suspected lymph node enlargement was described intraoperatively in two patients, while ascites was uncommon, observed in four cases in small volume and in two cases with larger effusions (1,000 mL and 1,700 mL). Surgical treatment was performed in nearly all cases, with total hysterectomy and bilateral salpingo-oophorectomy combined with complete lesion excision as the dominant approach. Lesions most frequently involved the rectosigmoid colon (60%, 34/57), followed by the vagina (19%, 11/57), ureter (18%, 10/57), bladder (7%, 4/57), liver (5%, 3/57), and stomach (1 case, 2%). Ovarian preservation was reported in 12 cases (21%), with a mean age of 45 years (range: 25–75). Pelvic lymphadenectomy and para-aortic lymphadenectomy were performed in 23 (40%) and 9 (16%) cases, respectively.

Histologically, endometrioid adenocarcinoma represented the most common subtype (35/57, 61.4%), followed by clear cell carcinoma (6/57, 10.5%), high-grade serous carcinoma (5/57, 8.8%), carcinosarcoma (3/57, 5.3%), mixed endometrioid and clear cell carcinoma (2/57, 3%), endometrial stromal sarcoma (2/57, 3.5%), adenosarcoma (1/57, 1.8%), squamous cell carcinoma (1/57, 1.8%), and mixed HGSC with large cell neuroendocrine carcinoma (1/57, 1.8%). One case showed histologic transformation from endometrioid adenocarcinoma to clear cell carcinoma at recurrence. Lymph node metastases were reported in pelvic (5 cases), para-aortic (1 case), and mesenteric nodes (2 cases). Molecular data remained limited, but KRAS mutations (2 cases), BRCA1 mutation (1 case), combined BRCA1 with MMR deficiency (1 case), MSH2/MSH6 alterations (1 case), MLH1 hypermethylation (1 case), and MMR-proficient status (2 cases) were reported.

Postoperative adjuvant chemotherapy was administered in 42 patients (73.6%). The most commonly used regimen was paclitaxel plus platinum (29 cases, 69.0%), followed by doxorubicin plus platinum (4 cases, 9.5%), cyclophosphamide plus platinum (3 cases, 5.3%), and cisplatin with 5-fluorouracil (2 cases, 3.5%). Targeted and emerging therapies were occasionally reported, including bevacizumab (3 cases, 7.1%), antibody–drug conjugates (1 case, 2.3%), and pembrolizumab (1 case, 2.3%). Radiotherapy was applied in 7 cases (16.7%), and progestin monotherapy in 3 cases (5.3%). During follow-up, disease progression occurred in two patients, stable disease in one, and recurrence in five cases.

## Discussion

Malignant transformation of DIE is often clinically silent until advanced, largely due to its deep pelvic location, fibrosis, and distortion of normal anatomical planes. The diagnostic delay has been reported to range from 4 to 11 years, and up to 65% of patients are initially misdiagnosed (57). In the present series, both patients developed hydronephrosis, a finding documented in only 17.5% of reported cases, suggesting it may be an under-recognized warning sign. Molecular profiling was rarely performed in previous reports (12.3%), whereas both of our cases underwent sequencing, identifying KRAS/PIK3CA mutations in Case 1 and KRAS alteration with HER2 2+ expression in Case 2. To our knowledge, this is the first report describing the use of a HER2-targeted antibody–drug conjugate in malignant DIE.

Several factors appear to contribute to malignant transformation, including hormonal exposure, chronic inflammation, immune dysregulation, and genetic alterations (58–60). Disease duration is likely relevant, as reflected by the mean 13.6-year interval observed in the reviewed cases. Clinically, symptoms remain indistinguishable from benign DIE, particularly dysmenorrhea, pelvic pain, and dyspareunia, which often delays recognition of malignant change. Ureteral or rectal involvement may manifest as hydronephrosis or bowel symptoms, yet these are frequently attributed to benign disease or managed in non-gynecologic departments, as seen in our cases.

Diagnosis requires integration of clinical findings, imaging, and laboratory markers. MRI and CT urography are particularly useful for evaluating deep pelvic invasion and urinary tract involvement, while PET/CT may provide additional metabolic information despite limited spatial resolution (61). CA-125 and HE4 are frequently elevated, with HE4 showing relatively better consistency across cases, suggesting potential value in risk stratification.

There are currently no standardized treatment guidelines. Surgical resection remains the cornerstone of management, typically requiring extensive pelvic procedures depending on disease extent (62). Although lymph node metastasis has been documented, its prognostic significance remains unclear, and the role of routine lymphadenectomy is still debated (63). Ovarian preservation may be feasible in carefully selected patients, but requires strict preoperative assessment and close follow-up (57).

Systemic chemotherapy, mainly paclitaxel plus platinum, remains the standard postoperative treatment (64). Immunotherapy has shown potential in endometriosis-associated malignancies with higher PD-L1 expression (65), but both of our cases showed low PD-L1 expression and low tumor mutational burden, suggesting limited benefit from immune checkpoint inhibition.

Antibody–drug conjugates represent an emerging therapeutic option. FRα-targeted agents are under investigation in gynecologic malignancies (66). In Case 2, HER2 expression (2+) provided a rationale for incorporating trastuzumab deruxtecan, highlighting the potential role of molecular stratification in treatment selection (67).

Both of our cases carried KRAS mutations, a finding also reported in prior literature (68). Multiple studies have detected cancer driver mutations in KRAS, PIK3CA, ARID1A, and PPP2R1A in the epithelial cells of benign endometriosis, including ovarian endometriomas and extraovarian DIE (69). About one-quarter of DIE patients harbor such mutations on comprehensive genomic analysis (70). Despite this prevalence, malignant transformation of extraovarian DIE is rare. KRAS and PIK3CA mutations may help endometriotic tissue implant and survive at ectopic sites, but they are not sufficient to cause cancer (24). In our Case 1, KRAS and PIK3CA mutations co-occurred. This combination occurs in 10-15% of endometriosis-associated ovarian cancers (EAOC) and activates both MAPK and PI3K/AKT pathways (71). In EAOC, the same mutations in ovarian endometriomas can lead to clear cell or endometrioid adenocarcinoma (70). Our two DIE cases progressed to malignancy with molecular profiles (KRAS, PIK3CA, wild-type p53, intact MMR) similar to EAOC, suggesting additional events may be needed for transformation. Candidate events include the PALB2 mutation in Case 1, which affects homologous recombination repair and may cause genomic instability, and HER2 2+ expression in Case 2. The SPOP mutation in Case 2 is less studied in gynecologic cancers; SPOP encodes a substrate-binding adaptor of the CULLIN3 E3 ubiquitin ligase complex and plays a crucial role in protein degradation through ubiquitination. In prostate cancer, loss-of-function SPOP mutations are an early event that stabilizes androgen receptors and increases AR signaling. In endometrial cancer, SPOP mutations alter substrate affinity, increasing degradation of bromodomain proteins while reducing degradation of estrogen receptors (72). The functional consequences of SPOP mutations in the context of DIE-associated malignancy remain unexplored, but given the estrogen-dependent nature of endometriosis, altered estrogen receptor signaling via mutant SPOP could theoretically contribute to malignant transformation (73). Both cases had wild-type p53 and intact MMR proteins, placing them in the NSMP category (60-76% of EAOC cases). Whether DIE-associated malignancies have distinct drivers or share the same molecular spectrum as EAOC requires larger studies.

Several limitations of this study should be noted. The relatively small sample size and possible publication bias may limit the generalizability of the findings. In Case 1, a direct histological transition from benign DIE to carcinoma was not demonstrated, although benign endometriotic tissue was seen in close proximity. The absence of a control group also limits comparison of tumor markers such as HE4. Follow-up time is still limited in some cases. In addition, there are currently no standardized diagnostic or treatment guidelines for this condition, and management is largely based on experience from ovarian cancer and expert consensus.

## Conclusions

The etiology of malignant transformation in DIE remains incompletely understood and is thought to involve genetic alterations, epigenetic changes, hormonal imbalance, and chronic inflammation. Based on our two cases together with 57 previously reported cases, several clinical observations can be made. Hydronephrosis was present in both of our patients but reported in only 17.5% of published cases, suggesting that it may be under-recognized in routine practice. In patients with long-standing DIE (≥10 years), new-onset or progressive hydronephrosis should raise concern for possible malignant transformation, particularly when accompanied by elevated HE4 levels. For follow-up, we propose a risk-adapted strategy, with surveillance every 6–12 months in stable patients and every 3–6 months in those with significant symptoms or larger lesions, incorporating symptom assessment, gynecologic examination, pelvic ultrasound, and serum CA-125 and HE4, while MRI, CTU, or PET/CT may be considered when clinically indicated. Molecular analysis in our cases revealed concurrent KRAS/PIK3CA mutations in Case 1 and KRAS alteration with HER2 2+ expression in Case 2; to our knowledge, this is the first report describing the use of an anti-HER2 antibody–drug conjugate in malignant DIE, suggesting that molecular profiling may help identify potential therapeutic targets in selected patients. Future multicenter studies with integrated multi-omics approaches, including whole-exome sequencing, single-cell analysis, and proteomics, are warranted to further clarify the mechanisms underlying malignant transformation and to facilitate the development of reliable biomarkers for early diagnosis and precision therapy.

## Data Availability

The original contributions presented in the study are included in the article/**Supplementary Material**. Further inquiries can be directed to the corresponding author.

## Supplementary material

Supplementary Figure 1 Clinical timelines of the two cases.

Supplementary Table 1 Characteristics of patients with malignant transformation of DIE.
