# Supplementary figures and images for: Case Report: Malignant transformation of deep infiltrating endometriosis: a report of two cases and literature review
Source: Front Oncol. 2026 Jun 18;16:1709462. doi: 10.3389/fonc.2026.1709462 (PMC13323627; doi:10.3389/fonc.2026.1709462)

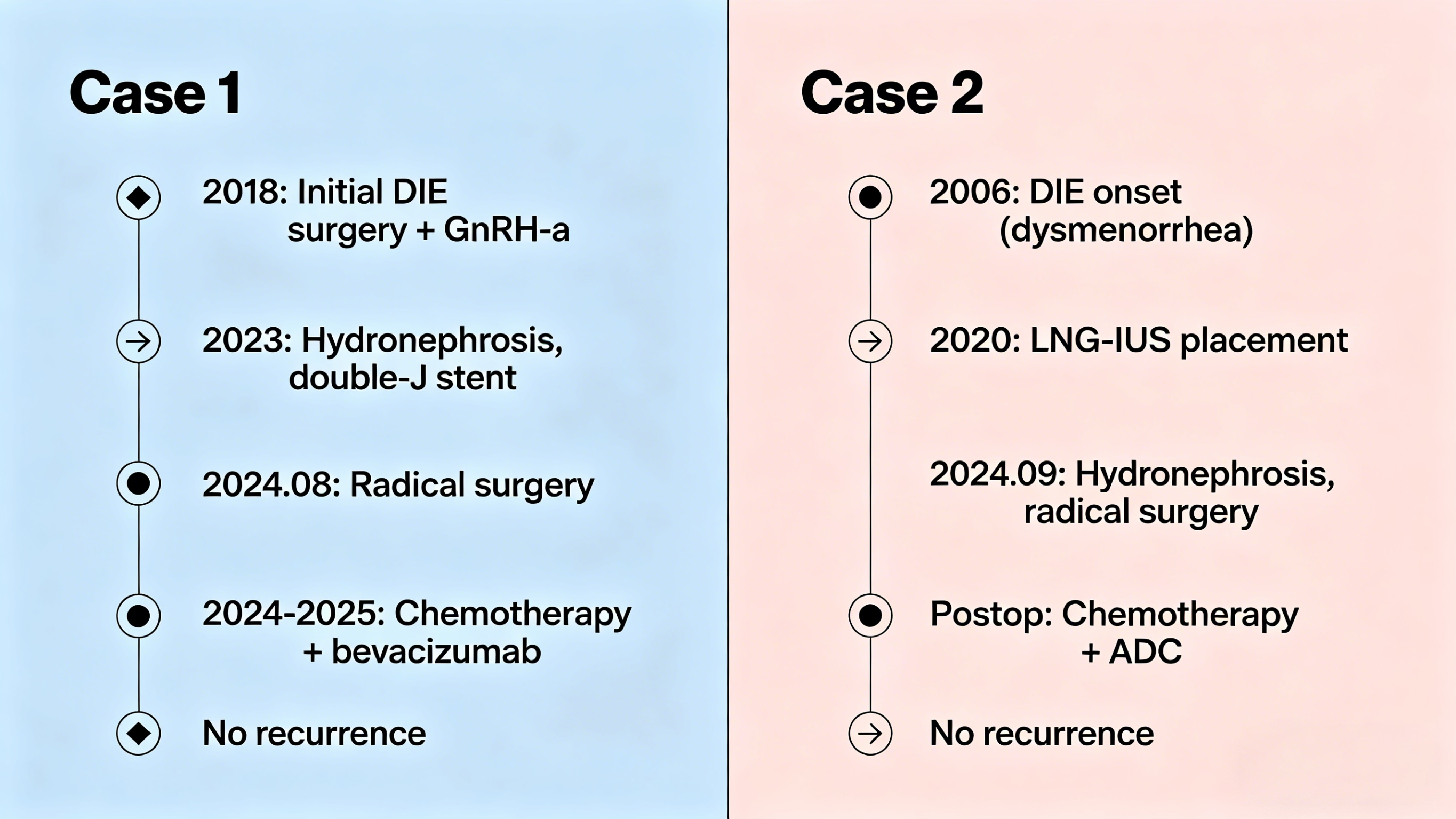

Supplement: Supplementary Figure 1 — Clinical timelines of the two cases. [file Image1.jpeg]
